# Efficacy of cx601 (darvadstrocel) for the treatment of perianal fistulizing Crohn’s disease—A prospective nationwide multicenter cohort study

**DOI:** 10.1007/s00508-023-02283-4

**Published:** 2023-10-12

**Authors:** Christopher Dawoud, Kerstin Melanie Widmann, Sascha Czipin, Michael Pramhas, Martina Scharitzer, Anton Stift, Felix Harpain, Stefan Riss

**Affiliations:** 1 Present Address: Department of General Surgery, Division of Visceral Surgery, Medical University Vienna, Waehringer Guertel 18–20, 1090 Vienna, Austria; 2 Department of Visceral, Transplant and Thoracic Surgery, Centre for Operative Medicine, Medical University of Innsbruck, Innsbruck, Austria; 3 First Surgical Department, Klinik Landstraße, Vienna, Austria; 4 Department of Biomedical Imaging and Image-Guided Therapy, Medical University Vienna, Vienna, Austria

**Keywords:** Perianal fistulizing Crohn’s disease, Stem cell therapy, Anal fistula, Fistula remission, Complex fistula

## Abstract

**Background:**

The use of mesenchymal stem cells is considered a novel and promising therapeutic option for patients with perianal fistulizing Crohn’s disease; however, data on its clinical application remain scarce. This multicenter nationwide study aimed to assess the clinical efficacy of mesenchymal stem cells in closing complex anal fistulas.

**Methods:**

In this study 14 Crohn’s disease patients (3 males, 11 females) with complex anal fistulas treated in 3 tertiary hospitals in Austria were included between October 2018 and April 2021.

Injection of 120 million allogeneic expanded adipose-derived mesenchymal stem cells (Cx601—darvadstrocel) was performed in each patient. Closure of the external fistula opening without secretion by external manual compression was defined as treatment success.

**Results:**

The median age of the patient population at the time of surgery was 32 years (range 26–53 years) with a median body mass index of 21.7 kg/m^2^ (range 16.7–26.6 kg/m^2^). Of the patients 12 (86%) received monoclonal antibodies (infliximab, adalimumab, ustekinumab, vedolizumab) at the time of surgery.

The median number of complex fistulas was 1.4 (range 1–2), The median operative time was 20 min (range 6–50 min) with no perioperative complications.

After a median follow-up of 92 weeks, we found successful fistula closure in 57.1% (*n* = 8) of treated patients. The perianal disease activity index did not improve significantly from initially 7 to a median of 6 after 52 weeks (*p* = 0.495).

**Conclusion:**

Darvadstrocel is a safe, minimally invasive surgical technique without significant perioperative complications. Clinical success can be expected in about half of the treated patients.

## Introduction

Perianal fistulas are common and occur in up to 40% of Crohn’s disease (CD) patients [1, 2]. In the first two decades after initial CD diagnosis, about 28% suffer from this complication [1], causing substantial morbidity and reduced the quality of life in those affected. The vast majority of fistulas in CD are classified as complex and are therefore difficult to treat as they are particularly refractory to conventional medical treatment strategies [3–5]. Furthermore, up to 70% of patients relapse after stopping conservative treatment, with only a few patients achieving long-term remission [6, 7].

Infliximab is currently the only approved anti-tumor necrosing factor (TNF) for fistulizing CD, which has shown efficacy in a randomized controlled clinical study [8]; however, after 1 year of treatment with infliximab, complete closure of draining fistula was demonstrated in only 23% of patients [9].

Although several surgical procedures to treat complex CD fistulas exist, they are associated with an increased risk of recurrence and incontinence [10].

As a new therapeutic option, allogeneic-expanded adipose-derived mesenchymal stem cells (Cx601) were introduced to manage perianal fistulizing CD. Mesenchymal stem cells are considered to promote anti-inflammatory and immunomodulatory effects and thereby support fistula healing [11, 12].

A placebo-controlled prospective phase III study by Panés et al. reported the efficacy of allogeneic adipose-derived mesenchymal stem cells in 51% of CD patients treated with Cx601-darvadstrocel [13], compared to 36% in the placebo group. Notably, further clinical data are still lacking, thus, its definite clinical efficacy still needs to be confirmed.

Therefore, the purpose of the present prospective nationwide study was to evaluate the efficacy of darvadstrocel in clinical practice.

## Methods

### Patients and data collection

In a nationwide prospective observational study, we enrolled patients with CD refractory to standard treatment for complex perianal fistula at three tertiary hospitals. We included adult patients aged 18 years and older who had a non-active or mildly active luminal CD with complex anal fistula with a maximum of two internal and three external fistula openings.

Patients were excluded if they had rectovaginal fistulas, rectal and/or anal stenosis, active proctitis, diverting stomas, and/or an abscess (abscess collection > 2 cm) that was not drained adequately at the fistula preparation visit.

Every patient underwent a pelvic magnetic resonance imaging (MRI) before surgery. Follow-up visits were scheduled for postoperative week 12, week 26 and week 52. At baseline, demographic data (age in years, gender [m/f], body mass index [BMI]) and medical history focusing on CD treatment were recorded. All patients were examined at baseline and every follow-up visit. Additionally, the perianal disease activity index (PDAI) and the Harvey Bradshaw index (HBI) were assessed. Medical treatment and changes in CD-specific immunomodulatory treatment were documented at each visit.

The study protocol was performed according to the ethical guidelines of the Declaration of Helsinki 1975, as the ethics committee of the Medical University of Vienna approved this study (#1682/2018). All patients gave written informed consent. Study registration: this study was registered at ClinicalTrials.gov (NCT05322057). This work has been reported in line with the STROCSS criteria [14].

### Surgical procedure

The procedure started with a seton removal, followed by the curettage of the fistula tract(s) and the internal fistula opening. The external fistula opening was excised, and the inner fistula opening was closed with an absorbable suture. The first 2 vials were injected around the internal fistula opening, the remaining 2 vials were administered around the fistula tract (injection of 120 million stem cells suspended in 24 mL).

### Outcome measurements

**Fig. 1:**
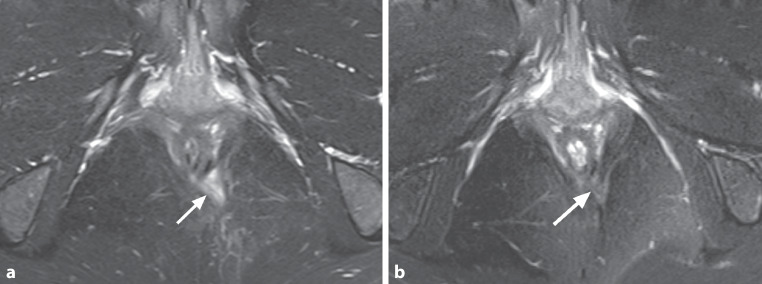
MRI of a 32-year-old female patient with perianal fistula. **a** Axial T2-weighted fat-saturated image before surgery shows a left-sided transsphincteric fistula with high signal (*arrow*), proving the fluid component within the fistula. **b** The fistula tract 10 months after surgery shows a dark signal on T2-weighted fat-saturated axial image (*arrow*), indicating fibrosis and response to treatment

The study primary endpoint was defined as fistula closure rate at postoperative week 52. Fistula healing was defined as closure of all treated external openings, no secretion after external finger compression and no signs of inflammation. Whenever possible, a pelvic MRI was conducted to further demonstrate fistula closure and to visualize a potential abscess formation (see Fig. 1).

### Statistical analysis

Statistical analysis was performed using SPSS statistical software package (IBM SPSS Statistics for Mac, Version 22.0). Continuous variables are expressed as median and range, as appropriate; categorical variables are presented as numbers with a percentage in brackets. Missing values are reported as unknown. Univariate analysis was performed using Student’s t‑test to explore quantitative variables and χ^2^-test if dichotomous. A *p*-value < 0.05 was considered to denote statistical significance.

## Results

Between 2018 and 2021, 14 patients (3 male, 11 female) were prospectively enrolled to the study and treated with an injection of darvadstrocel in 3 different tertiary hospitals in Austria and 21 patients did not meet the inclusion criteria and were excluded. The most common reasons for exclusion were rectovaginal fistulas, stomas and anal abscesses larger than 2 cm.

One patient underwent repetitive stem cell therapy due to failure after the first treatment session. The time interval between the 2 operations was 23 months.

Demographic data are outlined in Table 1. The median duration of an active anal fistula disease was 47 months (range 23–109 months).

**Table 1:** Demographics and baseline characteristics

	*n* = 14
**Demographics**	
*Age years, median (range)*	32 (26–53)
*Female sex, n (%)*	11 (79)
*BMI (kg/m*^*2*^*), median (range)*	21.7 (16.7–26.6)
**Clinical history**	
*CD diagnosis years (years), median (range)*	19 (9–30)
*Disease location*	
Ileal [L1], *n* (%)	7 (50)
Colonic [L2], *n* (%)	2 (14)
Ileocolonic [L3], *n* (%)	5 (36)
*Disease behavior*	
Stricturing [B2], *n* (%)	2 (14)
Penetrating [B3], *n* (%)	12 (86)
*History of smoking, n (%)*	3 (21)
Active smokers, *n* (%)	2 (14)
*Hospitalization in last 2 years due to Crohn’s disease, n (%)*	12 (86)
**Surgical history due to Crohn’s disease**	
*Prior surgical treatment, n (%)*	14 (100)
*One anal surgery, n (%)*	7 (50)
*Two anal surgeries, n (%)*	3 (21)
*More than three anal surgeries, n (%)*	4 (29)
**Conservative treatment at surgery**	
*Immunosuppressive therapy, n (%)*	13 (93)
*Monoclonal antibodies, n (%)*	12 (86)
Duration of therapy prior to surgery (months), median (range)	46 (6–96)

All fistulas showed a transsphincteric course and were treated with seton drainage prior to stem cell therapy. The mean number of anal fistulas treated per patient was 1.4 with a maximum of 2 fistulas.

The median operation time was 20 min (range 6–50 min). The median length of hospital stay was 2 days (range 2–3 days). We observed neither intraoperative nor postoperative complications.

The follow-up ranged from 52 to 156 weeks (median 92.0 weeks).

We observed 8 patients (57.1%) with complete fistula healing, demonstrated by a closure of the external opening and no secretion by external compression. One patient required two injections of darvadstrocel after initial treatment failure. The other patients, who did not show successful fistula closure, received a reapplication of a loose seton.

Due to incomplete MRI data, no systematic evaluation was performed.

The immunomodulatory medication was changed during the follow-up period in 4 patients (40%) to another biological treatment. None of those patients showed fistula healing after darvadstrocel injection.

**Fig. 2:**
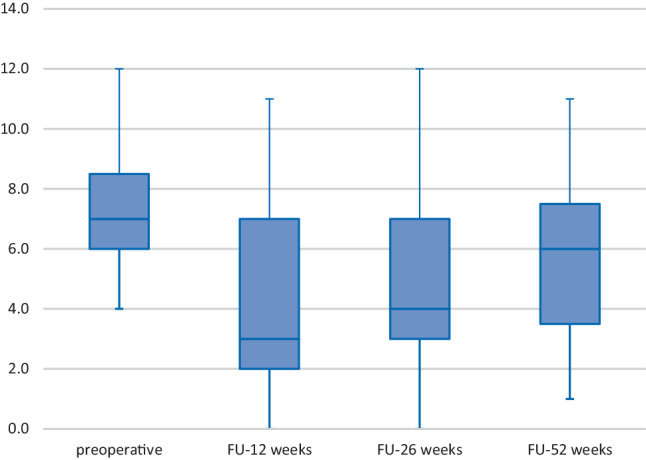
Box plot showing the course of PDAI from preoperatively to follow-up. *PDAI* perianal disease activity index, *FU* follow-up

The PDAI changed significantly at weeks 12 and 26 compared to the baseline scores (see Fig. 2) (baseline to week 12: *p* = 0.042; baseline to week 26: *p* = 0.039); however, PDAI did not change significantly from baseline to week 52 (baseline to week 52: *p* = 0.495).

**Fig. 3:**
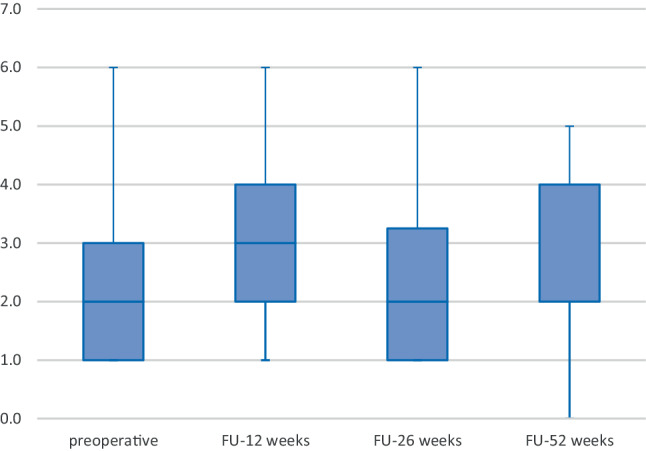
Box plot showing the course of the Harvey-Bradshaw index from preoperatively to follow-up. *FU* follow-up

The postoperative HBIs did not show a significant difference to the preoperative results (see Fig. 3) (baseline to week 12: *p* = 0.655; baseline to week 26: *p* = 0.564; baseline to week 52: *p* = 0.739).

## Discussion

Complex perianal fistulas are challenging to treat and debilitating for those patients who are affected. The main goal of surgical treatment is to achieve long-term fistula closure by preserving anal sphincter function. The finding of our nationwide cohort study showed that in a difficult to treat patient population with refractory complex perianal fistula, a 57.1% healing rate was achieved using allogeneic-expanded adipose-derived mesenchymal stem cells in combination with immunomodulatory medication. Additionally, our data confirmed the safety of this treatment with no observed serious adverse events in the perioperative period. Noteworthy, one patient developed an early abscess at the surgical site soon after the operation requiring incision with new placement of a loose seton. We defined this as an early treatment failure, rather than a side effect following stem cell application.

As the exact mechanism of mesenchymal stem cells in treating Crohn’s disease remains unknown, it is believed that due to their intrinsic immunomodulatory properties, a reduction of exacerbated inflammation can be achieved [15]. The immunomodulatory characteristics of mesenchymal stem cells have already found application in treatment of various diseases such as multiple sclerosis, graft-versus-host disease, myocardial infarction and Crohn’s disease [16–19]. The attributes of the cells result from three significant steps: migration to sites of active inflammation/tissue injury, secretion of anti-inflammatory molecules and paracrine signalling to nearby cells to maintain the local anti-inflammatory environment [15, 20–24].

The first successful treatment with mesenchymal stem cells was reported by Garcia-Olmo et al. [25]. A patient with a refractory rectovaginal fistula due to CD was treated by autologous stem cell transplantation with a lipoaspirate as the source of stem cells. After a follow-up of 3 months, the rectovaginal fistula appeared to be healed.

Notably, there is a lack of studies evaluating the role of perianal fistula remission after darvadstrocel therapy. Our fistula closure rate was in line with the results of the ADMIRE trial [13, 26]. In addition, we observed a significant improvement of the PDAI until the second postoperative control, while the HBI remained unchanged.

Cabalzar-Wondberg et al. included 11 patients with Crohn’s disease and described a healing rate of 72.7% after a median of 41.5 weeks (range 12–81 weeks) [27]. The short follow-up period and the missing radiological data could explain the surprisingly increased healing rate in contrast to the literature.

However, Schwandner reported in a single-centre cohort study a similar fistula healing rate of 66.7% in 12 CD patients treated with darvadstrocel [28]. The internal openings were closed by simple suture and there was one patient in which a mucosal flap was created to close the internal fistula opening before administration of the stem cells.

Nikolic et al. assessed the efficacy of darvadstrocel in the treatment of rectovaginal fistula in 4 patients after a follow-up of 6 months. Noteworthy, they reported a successful fistula healing in one patient (25%) only [29]. None of the patients had a stoma at the time of surgery which could explain the disappointing outcome. Further studies are necessary to clarify the role in this specific group of affected patients.

A phase I study by Dietz et al. investigated autologous mesenchymal stem cells applied in a bioabsorbable matrix to treat perianal fistulizing Crohn’s disease [30]. After 6 months, 10 of 12 patients (83%) showed clinical healing.

Patient selection is crucial to achieving optimal clinical outcomes. So far, the ideal patient for stem cell therapy has not been identified. Thus, we still need to obtain more data to predict treatment outcome more accurately. The use of concomitant immunosuppressive therapies, the grade of proctitis or the number of existing fistula tracts remain important influencing parameters, which need to be addressed in future trials.

The main limitation of our study is the low number of patients; however, considering the high cost of the stem cell therapy and the lack of clinical data, we still believe that we can further provide important results to more clearly define the role of stem cells in perianal CD.

## Conclusion

The study demonstrated the feasibility and safety of the application of mesenchymal stem cells in CD patients with complex anal fistulas. Clinical fistula closure can be expected in about half of the patients, treated with darvadstrocel.
